# Draft Genome Sequence of Bacillus safensis Strain Sami, Isolated from Leaf Veins of *Ficus religiosa*

**DOI:** 10.1128/MRA.00827-19

**Published:** 2019-11-21

**Authors:** Ghulam Muhammad Sami Ullah Asif

**Affiliations:** a Hope Genomics, Lahore, Pakistan; Indiana University, Bloomington

## Abstract

Here, I report the draft genome sequence of a novel Bacillus safensis strain, Sami, isolated from leaf veins of Ficus religiosa. F. religiosa is a large tree native to the Indian subcontinent and Indochina. The draft genome of B. safensis is 3.67 Mb.

## ANNOUNCEMENT

Bacillus safensis is normally considered a rhizobacterium and is found in root nodules of members of the Fabaceae family of plants (1). B. safensis is considered a plant growth promoter (1). Here, I report the draft genome sequence of a novel *B. safensis* strain, Sami, which was isolated from Ficus religiosa leaf veins. This is a new habitat of *B. safensis* (2).

To isolate the organism, the first step was to remove all kinds of contamination on the outer surface of the leaves, as *B. safensis* resides inside the leaf veins. Leaves of F. religiosa were washed with ethanol and sodium hypochlorite. After the leaves were washed, they were dried with flame. Leaf veins were cut with a blade and streaked onto nutrient agar at 37°C for 24 h. To extract genomic DNA for whole-genome sequencing, bacterial culture was grown in tryptic soy broth at 37°C. DNA extraction was performed using Qiagen’s DNeasy UltraClean microbial kit, and DNA integrity was checked by using gel electrophoresis. Library construction was performed using Illumina Nextera XT DNA library prep kits. The final library was quantified by quantitative PCR (qPCR) and an Agilent Bioanalyzer. The insert size for libraries was 350 bp.

The Illumina NextSeq 500 platform was used for sequencing the paired-end library. A total of 8,980,391 reads were generated after sequencing was performed. The read length was 2 × 75 bp, and the mean quality (Q) score for the sequence reads was 30. MiSeq Reporter v3.0 software with default parameters was used for adapter trimming. Those sequences which had >90% sequence similarity to adapter sequences were trimmed. The mean length of sequence reads after quality trimming was 72 bp. These high-quality paired-end reads were assembled by using the Unicycler assembler v0.4.6.0 with default parameters (3). Contigs having less than 0.25 of the chromosomal depth were filtered out. For SPAdes assembly, the lowest k-mer size was 0.2, and the highest was 0.95 (expressed as fractions of read length). Contigs shorter than 100 bp were excluded from the FASTA file.

Unicycler generated 30 contigs with a total length of 3,672,548 bp and an *N*_50_ value of 528,055. The mean sequence coverage depth of the final genome assembly was 30×. A total of 3,692 protein-coding regions were predicted using the NCBI Prokaryotic Genome Annotation Pipeline (4). One possible CRISPR locus was also predicted using CRISPRfinder (5; see also http://crispr.i2bc.paris-saclay.fr/). The GC content is 41.6% (6). After a genome BLAST search of the NCBI database, comparison with closely matching species was drawn using average nucleotide identity (ANI) values (7; see also http://enve-omics.ce.gatech.edu/ani). During ANI calculation, a window size of 1,000 bp and a step size of 200 bp were selected for fragment options. ANI values determined that *B. safensis* FO-36b (GenBank accession number CP010405), with an ANI value of 97.5%, is the closest species whose genome is available.

### Data availability.

This sequence was submitted to NCBI GenBank under accession number CP032830. Raw sequence reads were also deposited in the NCBI Sequence Read Archive under accession number SRR8181849.

## References

[1] Kothari VV, Kothari RK, Kothari CR, Bhatt VD, Nathani NM, Koringa PG, Joshi CG, Vyas BRM 2013 Genome sequence of salt-tolerant *Bacillus safensis* strain VK, isolated from saline desert area of Gujarat, India. Genome Announc 1:e00671-13. doi:10.1128/genomeA.00671-13. 24009116 PMC3764411

[2] Lateef A, Adedayo Adelere I, Gueguim-Kana EB 2015 The biology and potential biotechnological applications of *Bacillus safensis*. Biologia 70:411–419. doi:10.1515/biolog-2015-0062. PMC4684068 26740788

[3] Wick RR, Judd LM, Gorrie CL, Holt KE 2017 Unicycler: resolving bacterial genome assemblies from short and long sequencing reads. PLoS Comput Biol 13:e1005595. doi:10.1371/journal.pcbi.1005595. 28594827 PMC5481147

[4] Tatusova T, DiCuccio M, Badretdin A, Chetvernin V, Nawrocki E, Zaslavsky PL, Lomsadze A, Pruitt KD, Borodovsky M, Ostell J 2016 NCBI Prokaryotic Genome Annotation Pipeline. Nucleic Acids Res 44:6614–6624. doi:10.1093/nar/gkw569. 27342282 PMC5001611

[5] Grissa I, Vergnaud G, Pourcel C 2007 CRISPRFinder: a Web tool to identify clustered regularly interspaced short palindromic repeats. Nucleic Acids Res 35:W52–W57. doi:10.1093/nar/gkm360. 17537822 PMC1933234

[6] Zhou H-Q, Ning L-W, Zhang H-X, Guo F-B 2014 Analysis of the relationship between genomic GC content and patterns of base usage, codon usage and amino acid usage in prokaryotes: similar GC content adopts similar compositional frequencies regardless of the phylogenetic lineages. PLoS One 9:e107319. doi:10.1371/journal.pone.0107319. 25255224 PMC4177787

[7] Goris J, Konstantinidis KT, Klappenbach JA, Coenye T, Vandamme P, Tiedje JM 2007 DNA–DNA hybridization values and their relationship to whole-genome sequence similarities. Int J Syst Evol Microbiol 57:81–91. doi:10.1099/ijs.0.64483-0. 17220447

